# Preparation of Bismuth Vanadates with Rich Oxygen Vacancies Using Different Sol pH and Their Photocatalytic Behavior in Degradation of Methylene Blue

**DOI:** 10.3390/gels11080625

**Published:** 2025-08-09

**Authors:** Shengli Chen, Yuanyuan Zhong, Jie Yang, Daixiong Yang, Dong Liu, Xiaodong Zhu, Lin Huang

**Affiliations:** 1 School of Mechanical Engineering, Chengdu University, Chengdu 610106, China; 17208283620@163.com (S.C.); suzyy605@163.com (Y.Z.); yangdaixiong1998@163.com (D.Y.); xiaodangjia21@126.com (X.Z.); 2 Department of Basic Courses, Officers College of the People’s Armed Police Force, Chengdu 610218, China; sylviataro@163.com; 3 Sichuan Tuomai Xingzhong Technology Co., Ltd., Chengdu 610106, China; liudong20212025@163.com; 4 Sichuan Province Engineering Technology Research Center of Powder Metallurgy, Chengdu 610106, China

**Keywords:** photocatalyst, bismuth vanadate, pH control, methylene blue

## Abstract

Gel precursors were formed by reacting bismuth nitrate pentahydrate, acetic acid, sodium metavanadate, and NaOH. pH was adjusted using NaOH solution followed by calcination to obtain bismuth vanadate (BiVO_4_) photocatalysts. During synthesis, pH directly influenced the formation and structure of the gel network. Therefore, the effects of pH on the microstructure and photocatalytic activity of BiVO_4_ were investigated. At pH 3, the sample consisted of microspheres formed by tightly packed small particles. At pH 5, the microspheres transformed into aggregated flakes. Photocatalytic performance was evaluated through methylene blue (MB) degradation, revealing the sample prepared at pH 7 (7-BVO) demonstrated the highest efficiency. The electronic band structure, bandgap, and band edge positions of 7-BVO were probed by density functional theory (DFT) and UV-vis absorption spectra. Furthermore, photoluminescence spectroscopy, electrochemical measurements, active species trapping experiments and liquid chromatography mass spectrometry technique collectively revealed the possible mechanistic pathways for MB photodegradation by 7-BVO.

## 1. Introduction

With the rapid development of the economy, pollution has become an increasingly prominent concern. Since water is essential for human life, the effective treatment of water pollutants is critical to ensuring safe water supplies [1,2,3,4]. Common water treatment methods include physical, chemical, and biological approaches [5,6,7,8]. Among these, semiconductor photocatalysis has gained significant attention due to its green, environmentally friendly nature, ability to harness solar energy, and lack of secondary pollution [9,10,11].

Bismuth-based materials have emerged as a research focus due to their high solar energy utilization efficiency [12,13,14]. Specifically, bismuth vanadate (BiVO_4_) has attracted attention for its narrow bandgap and unique layered structure, which promotes charge separation [15,16,17,18,19]. Nguyen et al. [15] prepared BiVO_4_ employing 2-methoxyethanol (MeOH), ethylene glycol (EDOH), and glycerol (PTOH) as solvents. They demonstrated that the solvent environment influenced BiVO_4_ crystal morphology, producing spindle-shaped crystals in EDOH and PTOH, and rod-shaped crystals in MeOH. Dhakal et al. [16] synthesized Er^3+^/Yb^3+^-doped BiVO_4_ using Bi(NO_3_)_3_·5H_2_O, NH_4_VO_3_, Yb(NO_3_)_3_·5H_2_O, Er(NO_3_)_3_·5H_2_O. By adjusting the doping ion concentration, they reduced the bandgap of BiVO_4_. Fluorescence lifetime measurements revealed that Er^3+^/Yb^3+^ doping prolonged the excited-state lifetime of BiVO_4_.

Substantial evidence confirms that heat treatment temperature significantly influences phase transitions in photocatalysts [20,21,22,23]. Baral et al. [22] studied the phase evolution of BiVO_4_ synthesized at heat treatment temperatures ranging from 300 °C to 600 °C. At 300 °C, the product was tetragonal BiVO_4_; between 400 °C and 550 °C, a tetragonal/monoclinic mixed phase was observed, and monoclinic BiVO_4_ formed at 600 °C. pH exerts significant regulatory control over the morphology and structure of photocatalytic materials [24,25,26,27]. Lu et al. [25] synthesized BiOBr photocatalysts at varying pH levels of 1, 3, 5, 7. Results demonstrate that grain size decreased with increasing pH. At pH 7, the catalyst exhibited a flower-like microstructure and optimal photocatalytic activity, achieving a BPA degradation rate exceeding 95% after 5-h irradiation.

In summary, both pH and calcination temperature critically determine BiVO_4_ properties. Crucially, pH directly governs the architecture of gel networks, thereby mediating the microstructure and photocatalytic activity. Consequently, hydrated gel precursors were synthesized from Bi(NO_3_)_3_·5H_2_O, NaVO_3_, acetic acid, and NaOH. Crystalline BiVO_4_ was fabricated at varying pH (3–11) under isothermal calcination. pH-dependent crystallographic and microstructural evolution was investigated. The effects of pH on crystal structure and microstructure were investigated. Photocatalytic performance was evaluated using MB solution degradation. Density functional theory (DFT), fluorescence spectrum, UV-vis absorption spectroscopy, reactive species experiments, and electrochemical measurements were used to investigate the electronic band structure, the extent of electron-hole pairs recombination, the light response range, and the main active species in the photodegradation.

## 2. Results and Discussion

### 2.1. Crystal Structure and Chemical Bond

**Figure 1:**
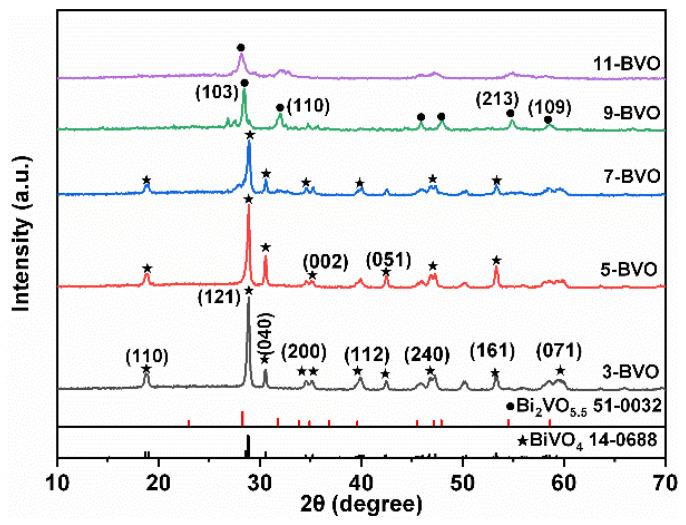
XRD patterns of 3-BVO, 5-BVO, 7-BVO, 9-BVO and 11-BVO (3-BVO, 5-BVO, 7-BVO, 9-BVO, and 11-BVO denote BiVO_4_ samples prepared under pH conditions of 3, 5, 7, 9, and 11, respectively).

Figure 1 shows the XRD patterns of samples prepared at different pH values (pH = 3, 5, 7, 9, 11). At pH 3 and 5, characteristic diffraction peaks appear at 18.7°, 28.8°, and 30.6°, corresponding to the (110), (121), and (040) crystal planes of monoclinic scheelite BiVO_4_ (m-s BiVO_4_) (PDF#14-0688) [17,28]. For pH 7, the intensity and position of these peaks are consistent with 3-BVO, confirming that the samples are m-s BiVO_4_ [18]. However, at pH 9, the intensity and position of the diffraction peaks change significantly, indicating a phase transformation. According to previous reports, BiVO_4_ reacts with OH^−^ under alkaline conditions to form Bi_2_VO_5.5_ [29,30]:

BiVO_4_ + OH^−^ → Bi_2_VO_5.5_ (1)

The diffraction peaks at 28.3° and 31.7° correspond to the (103) and (110) planes of tetragonal Bi_2_VO_5.5_ (PDF#51-0032), and no BiVO_4_ peaks is observed, confirming that 9-BVO is entirely tetragonal Bi_2_VO_5.5_. The peak intensity is lower than that at pH 3, 5, and 7, indicating reduced crystallinity. For 11-BVO, the diffraction peaks remain consistent with those of Bi_2_VO_5.5_, but with further reduced crystallinity. The calculated grain sizes for 3-BVO, 5-BVO, 7-BVO, 9-BVO, and 11-BVO are 35.7 nm, 28.7 nm, 21.3 nm, 24.0 nm, and 15.1 nm, respectively. These results indicate a decreasing trend in crystallinity and grain size with increasing pH.

**Figure 2:**
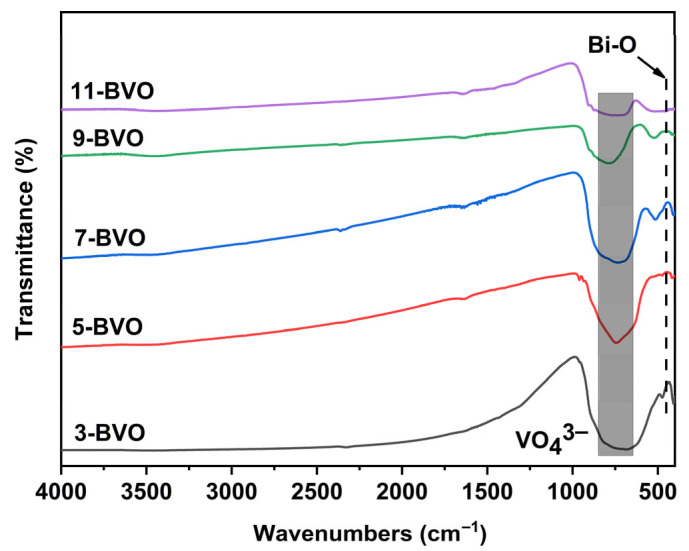
FT-IR spectra of 3-BVO, 5-BVO, 7-BVO, 9-BVO and 11-BVO.

Figure 2 exhibits the FT-IR spectra of BiVO_4_ samples prepared at different pH values. The peaks at 740 cm^−1^ and 840 cm^−1^ correspond to the asymmetric (v_3_) and symmetric (v_1_) stretching vibrations of the VO_4_^3−^ units [31]. The spectral feature at 520 cm^−1^ is assigned to the Bi–O stretching vibration [18].

### 2.2. Chemical Valence States Analysis

**Figure 3:**
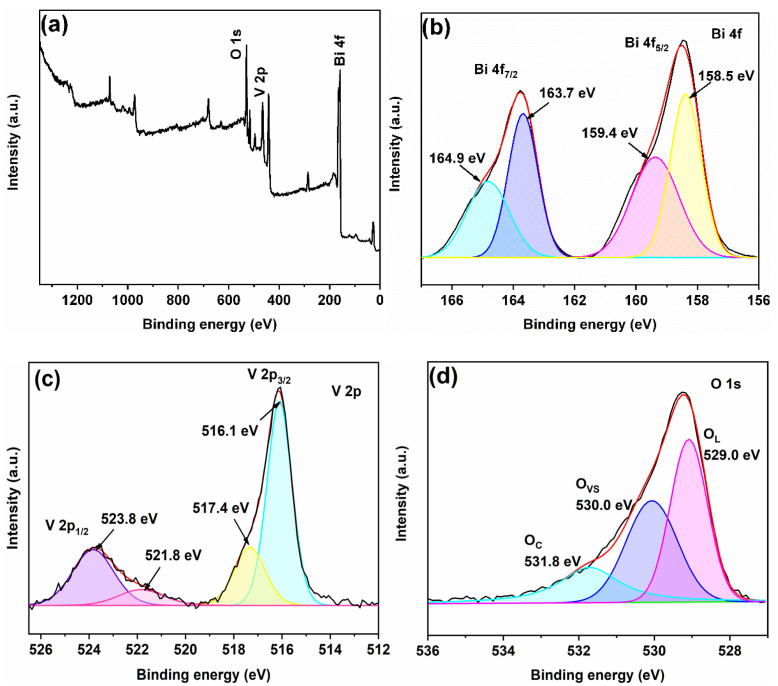
XPS spectra of 7-BVO: (**a**) total spectra; (**b**) Bi 4f; (**c**) V 2p; (**d**) O 1s.

XPS spectra of 7-BVO in Figure 3a confirm the sample consisting of Bi, V, and O. To further explore the chemical states of these elements, high-resolution spectra were obtained (Figure 3b–d). Two prominent peaks at 163.7 eV and 158.5 eV are attributed to Bi 4f_7/2_ and Bi 4f_5/2_, respectively, indicating the presence of Bi^3+^ [32,33]. Additionally, two minor peaks at 164.9 eV and 159.4 eV suggest the existence of Bi^(3+x)+^ valance. Studies indicate that Bi^3+^ can lose additional electrons to form Bi^(3+x)+^, a higher oxidation state with enhanced antioxidative properties. This high-valent Bi prevents oxygen vacancies from being refilled by O atoms, thus stabilizing the oxygen vacancies [12,14]. The high-resolution V 2p spectrum shows for distinct peaks at 523.8 eV and 521.8 eV, 517.4 eV and 516.1 eV, corresponding to V 2p_1/2_ and V 2p_3/2_, respectively, indicating the presence of both V^4+^ and V^5+^ oxidation state [34,35,36]. The O 1s spectrum reveals three peaks. The peak at 529.0 eV corresponds to lattice oxygen, the peak at 531.8 eV is attributed to adsorbed oxygen, and the peak at 530.0 eV is associated with oxygen vacancies [14,37,38,39].

### 2.3. Morphology and Specific Surface Area Analysis

**Figure 4:**
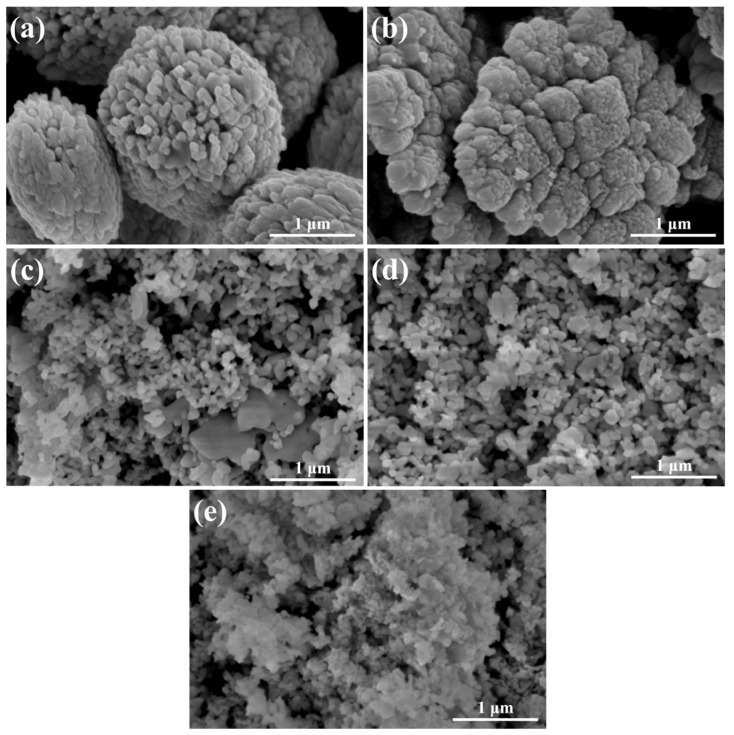
SEM images of samples at 100,000×: (**a**) 3-BVO; (**b**) 5-BVO; (**c**) 7-BVO; (**d**) 9-BVO; (**e**) 11-BVO.

SEM images of samples at different pH values are shown at 100,000× in Figure 4. 3-BVO exhibits microspherical agglomerates formed by densely packed small particles with sizes ranging from 2 to 3 µm. At pH 5, the microspherical agglomerates transition to sheet-like structures. The agglomerates of 7-BVO, 9-BVO, 11-BVO further disperse into smaller particles.

**Figure 5:**
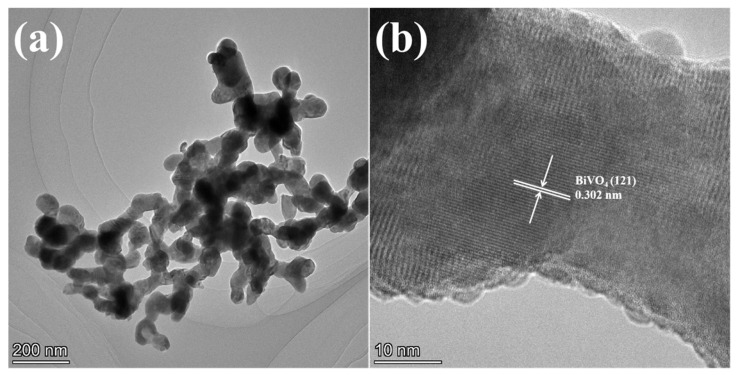
TEM (**a**) and HRTEM (**b**) images of 7-BVO.

The TEM image (Figure 5a) reveals that 7-BVO consists of dispersed rice-liked particles with sizes ranging from 50 to 80 nm. The HRTEM image (Figure 5b) shows uniform and well-defined lattice fringes, indicating high crystallinity. The measured lattice spacing of 0.302 nm corresponds to the (121) crystal plane of BiVO_4_ [31].

**Figure 6:**
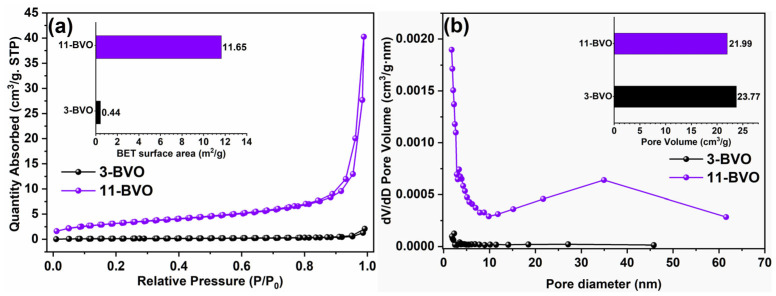
N_2_ adsorption desorption curves (**a**) and pore size distribution curves (**b**) of 3-BVO and 11-BVO.

The N_2_ adsorption-desorption isotherms and pore volume distribution curves for 3-BVO and 11-BVO are shown in Figure 6. In Figure 6a, the specific surface areas of 3-BVO, and 11-BVO are 0.44 m^2^/g and 11.65 m^2^/g, respectively, with 7-BVO at 7.79 m^2^/g [18], indicating an increasing trend with higher pH. The average pore volumes of 23.77 cm^3^/g for 3-BVO and 21.99 cm^3^/g for 11-BVO in Figure 6b, whereas 7-BVO (13.55 cm^3^/g) demonstrates significantly lower porosity [18]. According to the specific surface area (BET) analysis and micromorphology results, 3-BVO consists of tightly packed small particles, resulting in a lower specific surface area and fewer active sites for photocatalytic reactions. In contrast, 7-BVO and 11-BVO, with their dispersed small particles, exhibit larger specific surface areas.

### 2.4. Photocatalytic Performance Analysis

**Figure 7:**
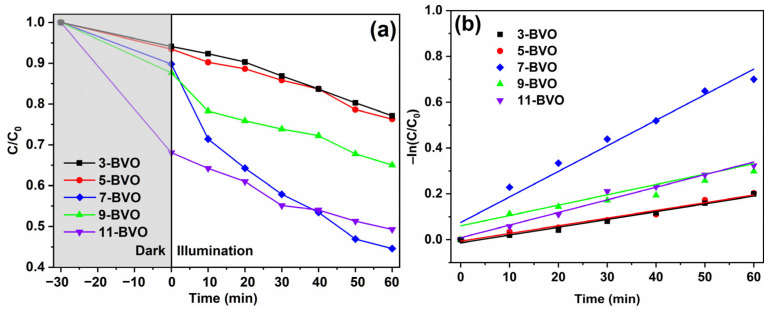
Photocatalytic degradation curves (**a**) and first order kinetic curves (**b**) of 3-BVO, 5-BVO, 7-BVO, 9-BVO and 11-BVO.

The photocatalytic performance was evaluated through measuring the degradation of MB under light irradiation, with results shown in Figure 7a. After 30 min of dark adsorption, the amounts of adsorbed pollutants increased with rising pH values. This trend likely results from the larger agglomerate size and denser particle packing of BiVO_4_ crystals synthesized at pH 3 and 5, leading to a smaller specific surface area and poorer adsorption performance. In contrast, BiVO_4_ prepared at pH 7, 9, and 11 exhibited smaller, more dispersed particles with larger specific surface areas, consistent with the BET results.

After 1 h of illumination, the degradation degrees for 3-BVO, 5-BVO, 7-BVO, 9-BVO, and 11-BVO were 22.9%, 23.7%, 55.4%, 35.0%, and 50.8%, respectively [18]. The photocatalytic reaction follows a first-order kinetic model described by –ln(C/C_0_) = kt, with the first-order kinetic fitting curves shown in Figure 7b. The highest photocatalytic activity was observed for 7-BVO, with a k of value of 0.0112 min^−1^ [18]. Samples prepared at pH 3 and 5 exhibited poor photocatalytic performance due to their smaller specific surface areas and limited reaction sites. At pH 9 and 11, the phase transition from m-s BiVO_4_ to tetragonal Bi_2_VO_5.5_ occurred, accompanied by reduced crystallinity and increased amorphous Bi_2_VO_5.5_. This phase transition led to a higher recombination rate of photogenerated electron-hole pairs, thereby reducing photocatalytic efficiency.

**Figure 8:**
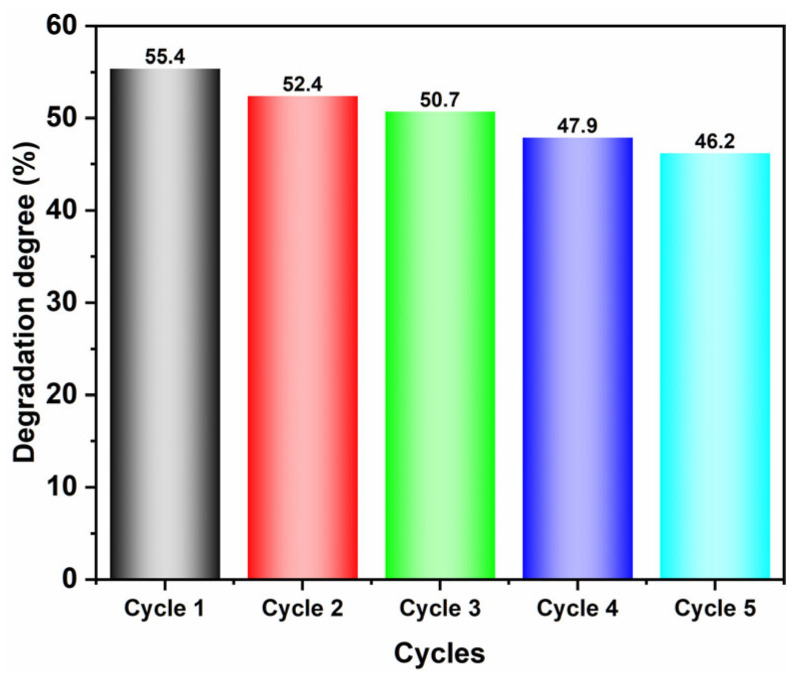
The five cyclic experiment results of 7-BVO for MB degradation.

The reusability of 7-BVO was assessed through five cyclic experiments, with results presented in Figure 8. After five cycles, the degradation efficiency of 7-BVO slightly decreased, likely due to the coverage of the photocatalyst sites [14].

**Figure 9:**
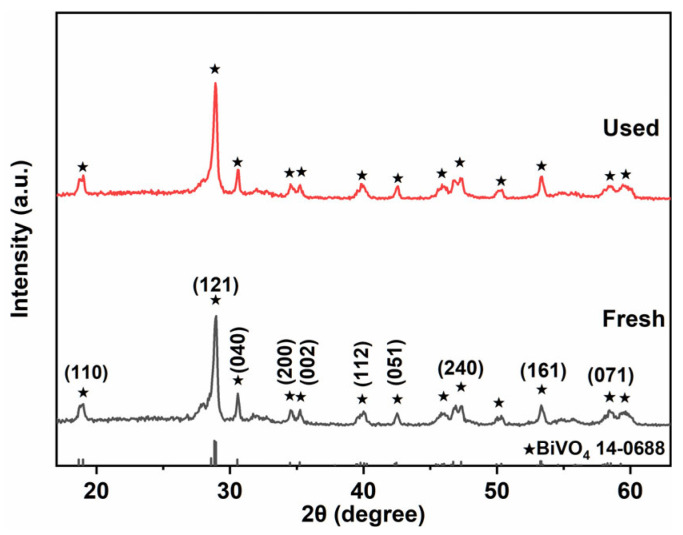
XRD patterns of 7-BVO before and after the five cyclic experiments.

XRD patterns of the sample before and after the photodegradation (Figure 9) showed no significant changes in the peak shape or position, indicating that 7-BVO retained its m-s BiVO_4_ structure throughout the reactions. This demonstrates the structural stability of the material during photocatalytic processes.

### 2.5. Photocatalytic Mechanism

**Figure 10:**
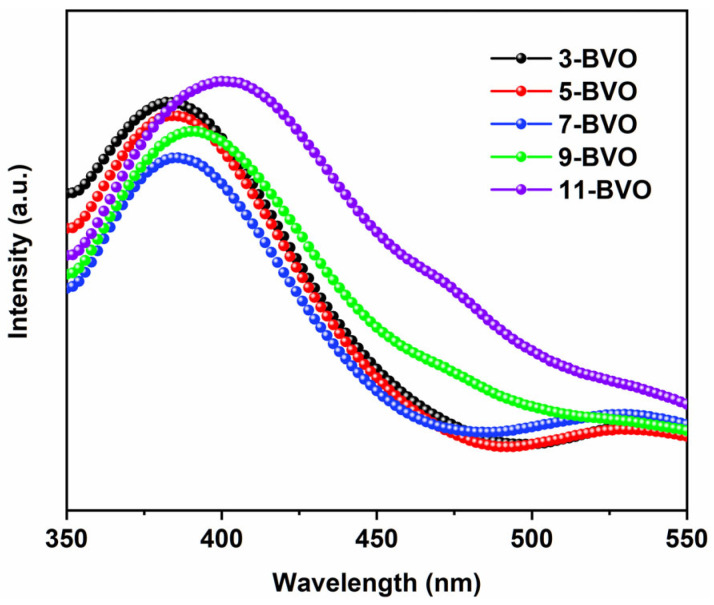
PL spectra of 3-BVO, 5-BVO, 7-BVO, 9-BVO and 11-BVO.

In PL spectra, higher PL peak intensity indicates a higher recombination rate, which reduces the number of photogenerated carriers available for redox reactions, thereby hindering photocatalytic activity [14,39]. As shown in Figure 10, the PL peak intensity decreased initially and then increased as the pH increased from 3 to 11, with the lowest PL intensity observed at pH 7 [18]. This indicates that 7-BVO exhibits the lowest recombination rate. XPS analysis revealed the presence of highly oxidizing Bi^(3+x)+^ valence in 7-BVO. These ions stabilize oxygen vacancies by preventing oxygen atoms from refilling the vacancies, thereby enhancing the separation efficiency of photogenerated photocarriers.

**Figure 11:**
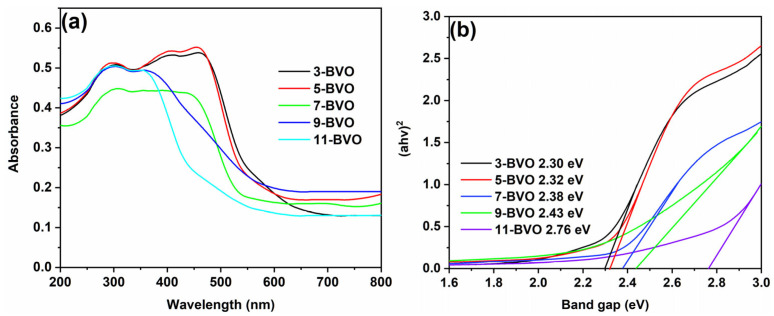
UV-vis absorption spectra (**a**) and band gaps (**b**) of 3-BVO, 5-BVO, 7-BVO, 9-BVO and 11-BVO.

The UV-vis absorption spectra shown in Figure 11a indicate that the light absorption range of the samples narrows with increasing pH, suggesting a reduced light response range. The band gaps (E_g_) were calculated using the Tauc relation [40]:

ahv = A (hv − Eg)1/n(2)

where h is Planck’s constant, v is the photon frequency, A is a proportional constant, and n depends on the type of semiconductor (2 for indirect and 1/2 for direct semiconductors). BiVO_4_, a direct semiconductor [29], showed band gap energies of 2.30 eV, 2.32 eV, 2.38 eV, 2.43 eV, and 2.76 eV for 3-BVO, 5-BVO, 7-BVO, 9-BVO, and 11-BVO, respectively [18] (Figure 11b).

**Figure 12:**
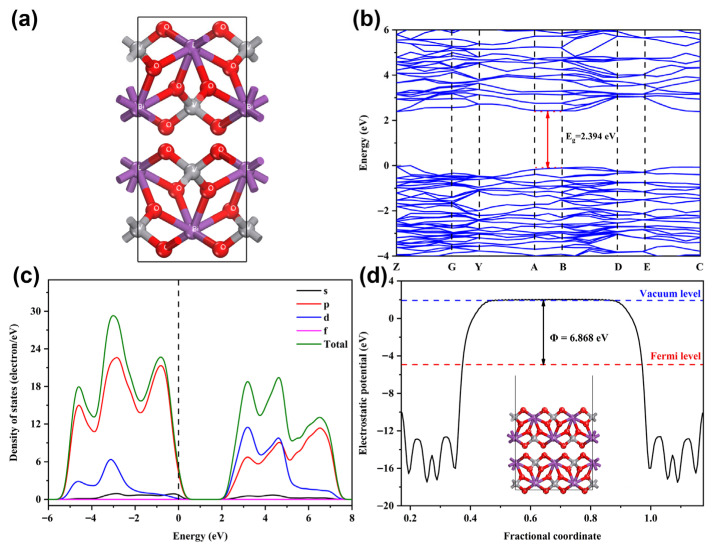
Optimized models (**a**), bandgap energies (**b**), density of states (DOS) (**c**) and work functions (**d**) of 7-BVO.

Density functional theory (DFT) calculations determined the band gap, density of states (DOS), and work function of 7-BVO (Figure 12). The optimized structure in Figure 12a exhibits a narrow band gap of 2.394 eV (Figure 12b), which closely matches the 2.38 eV value derived from Tauc plot analysis. As shown in Figure 12c, the valence band maximum (VBM) is predominantly composed of O 2p orbitals, while the conduction band minimum (CBM) is dominated by V 3d states. Figure 12d presents the work function (6.868 eV), quantifying the energy required for electron emission from the Fermi level to the vacuum level [41,42].

**Figure 13:**
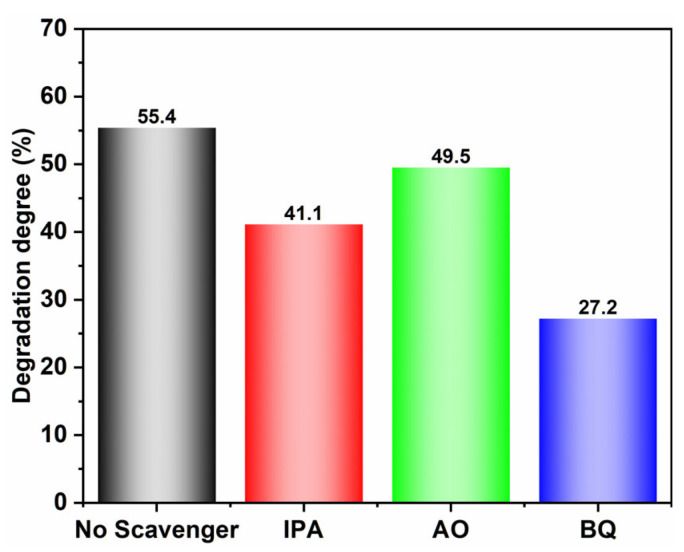
Active species experiment results of 7-BVO.

To identify the primary reactive species, various scavengers were introduced during the photocatalytic reaction of 7-BVO (Figure 13). Adding ammonium oxalate (AO) and isopropanol (IPA) had mild impact on the degradation of MB, indicating that photogenerated holes (h^+^) and hydroxyl radicals (·OH) were not the main reactive species. However, the addition of benzoquinone (BQ) significantly inhibited MB degradation, with the degradation rate dropping to 27.2%, suggesting that superoxide radicals (·O_2_^−^) are the primary reactive species.

**Figure 14:**
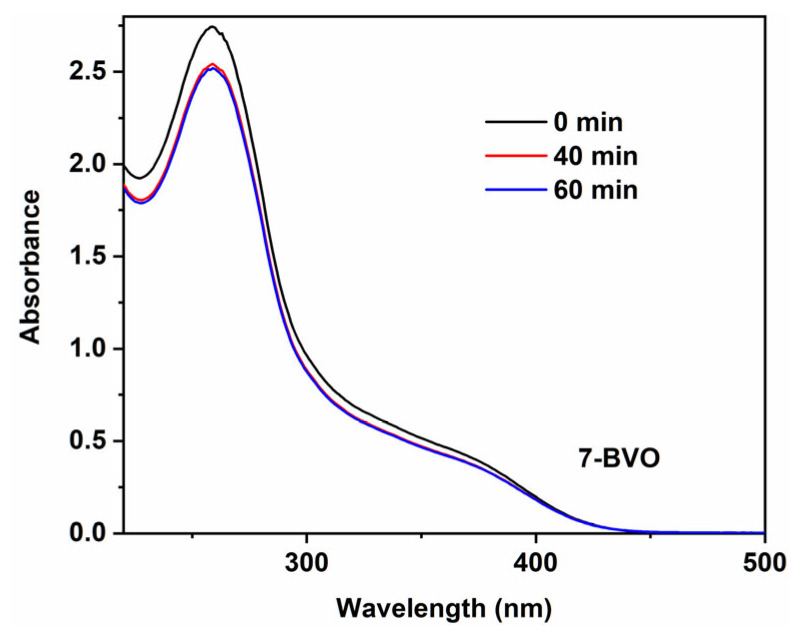
NBT absorbance curves of 7-BVO.

Nitroblue tetrazolium (NBT) experiments further confirmed the formation of ·O_2_^−^ during the photocatalytic process. As shown in Figure 14, the absorbance of NBT decreased with increased illumination time, indicating that ·O_2_^−^ reacted with NBT, producing a purple precipitate and consuming NBT in the process [43].

**Figure 15:**
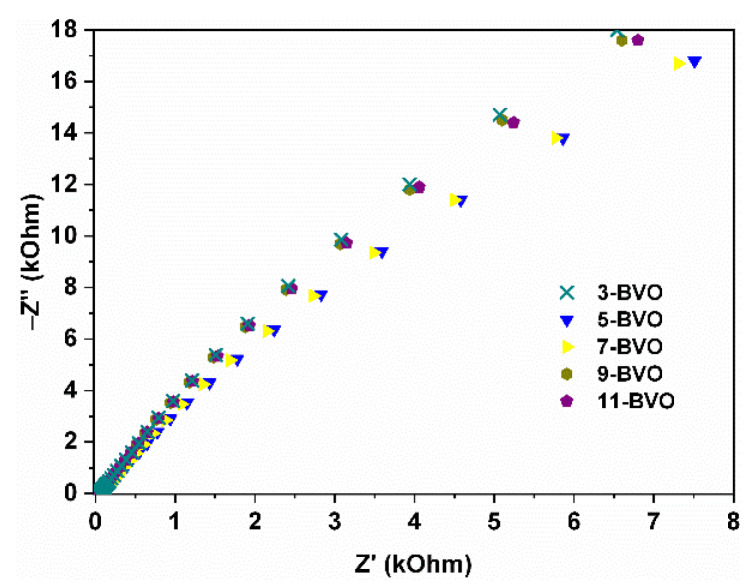
Electrochemical impedance spectroscopy of 3-BVO, 5-BVO, 7-BVO, 9-BVO and 11-BVO.

In electrochemical impedance spectroscopy (Figure 15), smaller semicircle radii for 7-BVO indicate lower charge transfer resistance [18], facilitating rapid migration of photogenerated carriers for photocatalytic degradation [11,39].

**Figure 16:**
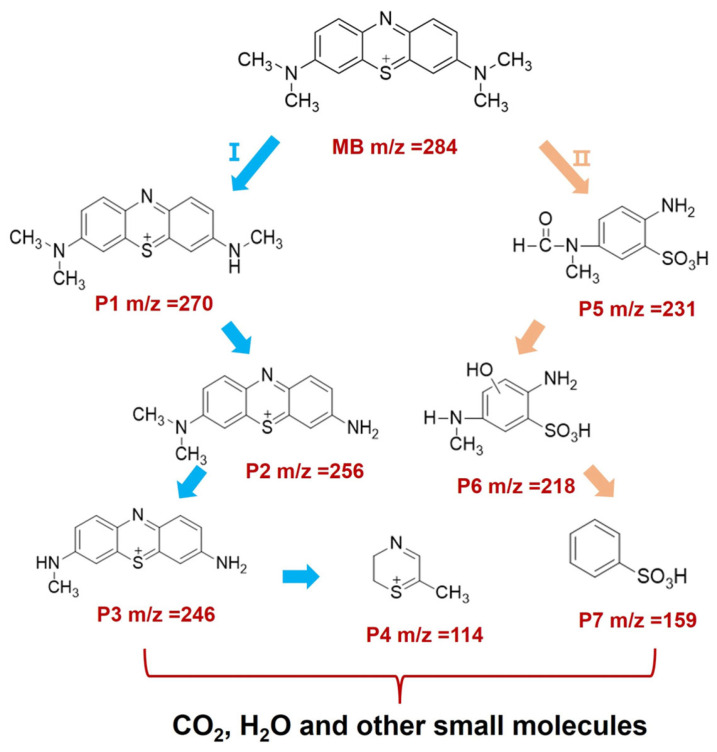
Possible degradation pathways of 7-BVO for MB.

The liquid chromatography-mass spectrometry technique was employed to analyze the main degradation pathways and intermediate products generated during the photocatalytic degradation of MB by 7-BVO. As shown in the Figure 16, MB may undergo two main degradation pathways. It undergoes chemical reactions under the action of ·OH, h^+^ and ·O_2_^−^, generating intermediate products with reduced molecular weight. Eventually, MB is decomposed into CO_2_, H_2_O and other small molecules [44,45,46].

## 3. Conclusions

Gel precursors were synthesized from bismuth nitrate pentahydrate, acetic acid, sodium metavanadate, and NaOH. Subsequent pH modulation (pH = 3, 5, 7, 9, 11) via NaOH solution and calcination yielded bismuth vanadate (BiVO_4_) photocatalysts. The pH-governed structure of three-dimensional gel networks influenced the microstructure and photocatalytic performance of BiVO_4_ At pH 3, the sample comprised tightly packed microspheres formed by small particles. At pH 5, the microspheres transitioned into sheet-like aggregates, while at pH 7, 9, and 11, the aggregates dispersed further into fine, granular particles. Samples prepared at pH 3, 5, and 7 exhibited a m-s BiVO_4_ phase, whereas at pH 9 and 11, m-s BiVO_4_ reacted with OH^−^, forming a tetragonal Bi_2_VO_5.5_ phase. The specific surface area increased with rising pH (3–11). XPS analysis confirmed the presence of highly oxidizing Bi^(3+x)+^ valance in 7-BVO, which prevented the refilling of oxygen vacancies by oxygen atoms, ensuring oxygen vacancy stability and enhancing charge carrier separation. DFT calculations reveal that in 7-BVO, VBM is predominantly composed of O 2p orbitals, while the conduction band minimum CBM is primarily dominated by V 3d states.PL spectra revealed that 7-BVO exhibited the lowest recombination rate of photocarriers. Photocatalytic experiments demonstrated that 7-BVO exhibited the highest photocatalytic activity, achieving a 55.4% degradation degree of MB after 1 h of irradiation. Active species trapping experiments identified ·O_2_^−^ as the dominant reactive species in the photocatalytic process.

## 4. Materials and Methods

### 4.1. Reagents

All reagents used in this experiment were of analytical grade. Sodium metavanadate, bismuth nitrate pentahydrate, glacial acetic acid, sodium hydroxide, 1,4-benzoquinone (BQ), ammonium oxalate (AO), isopropanol (IPA), methylene blue (MB), and nitroblue tetrazolium chloride (NBT), were purchased from Chengdu Kelong Chemical Co., Ltd. (Chengdu, China). Poly vinylidene fluoride (PVDF), was purchased from Zhengzhou Jinghong New Energy Technology Co., Ltd. (Zhengzhou, China), and N-Methyl-2-pyrrolidone (NMP), was purchased from Titan Scientific Co., Ltd. (Shanghai, China).

### 4.2. Synthesis of Photocatalytic Materials

Bismuth nitrate pentahydrate (5 mmol, 2.425 g) was dissolved in 50 mL of 1 M acetic acid to form a homogeneous bismuth-based precursor sol (Sol A), where acetic acid acted as both chelating agent and reaction medium to suppress premature Bi^3+^ precipitation. Simultaneously, sodium metavanadate (5 mmol, 0.61 g) was dissolved in 50 mL of 1 M NaOH solution, yielding a stable vanadate ion sol (Sol B). Under continuous stirring, Sol B was dropwise added to Sol A to initiate hydrolysis and condensation between Bi^3+^ and VO_4_^3−^ ions, establishing primary Bi-O-V linkages. The pH was subsequently adjusted to predetermined values (3, 5, 7, 9, 11) using 1 M NaOH-a critical parameter governing hydrolysis/polycondensation kinetics, colloidal surface charge, and sol stability that directly determined gel network formation. The mixture was then magnetically stirred at 80 °C for 60 min, enabling enhanced Bi-O-V network growth, particle aging/crosslinking, and viscosity increase that facilitated sol-to-gel transition, ultimately yielding a yellow suspension. Precipitates were collected by centrifugation, washed with deionized water to remove ionic residues, and dried to obtain xerogel precursors. Subsequent calcination at 400 °C for 1 h in a muffle furnace eliminated organic/hydroxyl species while converting amorphous networks to crystalline BiVO_4_. Final products were ground and labeled as x-BVO (x = 3, 5, 7, 9, 11) according to synthesis pH.

### 4.3. Characterization

The phase compositions were analyzed using a DX-2700 X-ray diffractometer (Dandong Haoyuan Instrument Co., Ltd., Dandong, China). Surface morphology was examined using an FEI-nspect F50 SEM (Boyue Instrument Co., Ltd., Shanghai, China) and an FEI Tecnai G2 F20 TEM/HRTEM (Fortress Energy Incorporated Company, Oregon, OR, USA). Elemental Analysis: Elemental composition and valence states were analyzed using an XSAM800 multifunctional surface analysis system (Kratos Analytical, Manchester, UK). Nitrogen adsorption-desorption data were recorded using a Micromeritics ASAP 2460 analyzer (Beijing Guanyuan Technology Co., Ltd., Beijing, China) to determine surface area and porosity. UV-vis absorption spectra were characterized using a UT-1950 double-beam UV-vis spectrophotometer (Puxi Instrument and Meter Co., Ltd., Shanghai, China). The rate of electron-hole pairs recombination was assessed using a Hitachi F-4600 fluorescence spectrophotometer (Techcomp Instrument Co., Ltd., Shanghai, China). Photocatalytic performance was evaluated using a DH7000 electrochemical workstation (Jiangsu Donghua Analytical Instruments Co., Ltd., Jingjiang, China).

### 4.4. Photocatalytic Activity Test

A 25 mg sample was added to 100 mL of a 15 mg/L MB solution. The mixture was stirred in the dark for 30 min to achieve adsorption equilibrium. 350 W xenon lamp (NBeT, Beijing, China) was then used to irradiate the solution for 1 h. During irradiation, 2 mL aliquots were taken every 10 min, centrifuged, and the absorbance at 664 nm was measured via UV-vis spectrophotometer.

### 4.5. Active Species Trapping Experiment

Ammonium oxalate (AO), benzoquinone (BQ), and isopropanol (IPA) were used to capture photogenerated holes (h^+^), superoxide radicals (·O_2_^−^), and hydroxyl radicals (·OH), respectively. For each experiment, 2 mL of a 2 mmol/L trapping agent was added to the photocatalytic system, keeping all other conditions constant. Results were compared with a control group to determine the active species involved.

### 4.6. NBT Reduction Test

25 mg of BiVO_4_ was dissolved in 100 mL of 5 mmol/L nitroblue tetrazolium (NBT) solution. The suspension was irradiated for 1 h, with samples taken every 10 min. After centrifugation, the supernatant was analyzed by UV-vis spectroscopy to measure changes in absorbance at 259 nm.

## Data Availability

Dataset available on request from the authors.
